# Damage in old leaves of shade-treated tea trees induced by high light after shade removal and shoot harvest

**DOI:** 10.5511/plantbiotechnology.25.0109a

**Published:** 2025-03-25

**Authors:** Shigeto Morita, Yuka Yanoh, Natsu Hamano, Mitsuhiro Nagata, Tetsuyuki Takemoto, Takehiro Masumura, Satoshi Sano

**Affiliations:** 1 Graduate School of Life and Environmental Sciences, Kyoto Prefectural University, Sakyo, Kyoto, Kyoto 606-8522, Japan; 2 Basic Research Division, Biotechnology Research Department, Kyoto Prefectural Agriculture, Forestry and Fisheries Technology Center, Seika, Soraku, Kyoto 619-0244, Japan; 3 Department of Agriculture, Forestry and Fisheries, Kyoto Prefectural Government, Kamigyo, Kyoto, Kyoto 602-8570, Japan; 4 Agriculture and Forestry Technology Department, Kyoto Prefectural Agriculture, Forestry and Fisheries Technology Center, Kameoka, Kyoto 621-0806, Japan

**Keywords:** *Camellia sinensis*, high light, oxidative stress, shading treatment

## Abstract

High-quality green tea is produced from developing shoots (apical buds and young leaves) of tea plants (*Camellia sinensis* (L.) Kuntze) grown under shaded conditions. However, the removal of shade covers causes shaded tea plants to experience a sudden exposure to high light (HL). Since in ordinary tea plantation new shoots are cropped immediately following shade removal, the remaining leaves emerging from the canopy are exposed to HL. In this study, we investigated the HL response of old leaves on shaded tea plants to evaluate possible deleterious effects of HL illumination after shade removal and shoot harvesting in two years (2017 and 2018). Old leaves of both shade-grown and unshaded tea plants suffered from temporal photoinhibition caused by HL exposure after shoot harvesting but were able to recover within two weeks. Moreover, chlorophyll *a*/*b* ratios remained unchanged in old leaves experiencing shading treatment, suggesting that old leaves have a weakened capacity to respond to low light conditions. Furthermore, protein carbonyl content was elevated 3–7 days after shade removal in summer 2018. Shoot growth during the subsequent autumn season was inhibited in shaded plants relative to the control group. Taken together these results indicate that old leaves on shaded tea plants suffer from oxidative damage after shade removal in summer, and this may inhibit the growth of autumn shoots.

The tea plant (*Camellia sinensis* (L.) Kunze) is an important commercial beverage crop whose apical buds and young leaves (developing shoots) are harvested and used for the production of tea. Tea is mainly consumed as green tea in Japan, and high-quality green teas, such as Matcha and Gyokuro, are produced from developing shoots grown under shaded conditions. Shade cultivation involves growing tea plants under low light (LL) conditions, where it increases the content of chlorophylls (Chls) and free amino acids (Ji et al. 2018; Ku et al. 2010; Lee et al. 2013; Liu et al. 2018; Sano et al. 2018; Wang et al. 2012), both of which contribute to deeper coloring and higher-quality taste of green tea. In Japanese tea plantations, tea trees generally produce new shoots three times a year. The harvest obtained from newly-emerging shoots that develop in April and May is called “the first crop”, while the one obtained in July is termed “the second crop”. Around Kyoto, shade cultivation has initially only been used during the first cropping season; however, over the last 20 years it has also been used for the second cropping season to meet increasing demand for high-quality green tea (Takemoto and Hayashi 2019). However, repeated shade cultivation during both the first and second cropping seasons over many years can negatively affect the growth of new shoots, thereby decreasing the yield of tea shoots (Takemoto and Hayashi 2019). The effects of repeated shade cultivation on tea plants include decreased starch and sugar content, increased leaf surface and canopy temperature, and increased citrulline and glycine betaine levels (Yamashita et al. 2020). Increased leaf surface and canopy temperature may reflect the suppression of transpiration and photosynthesis, and therefore represent an important negative effect of repeated shade cultivation. Another deleterious effect of shading treatment is the high light (HL) stress caused by the exposure of leaves to HL following shade removal. We previously found that tea plants grown under shaded conditions experienced temporal photooxidative stress after being exposed to HL illumination but were able to recover from oxidative damage within two weeks (Sano et al. 2020). We further revealed that HL stress in shade-treated tea plants could be alleviated by acclimation to light before shade removal (Morita et al. 2022).

In a previous study, we investigated the HL stress response in new shoots of shade-treated tea plants (Sano et al. 2020). Although new shoots are harvested immediately after shade removal in ordinary tea plantation, we did not crop new shoots at the end of the shading treatment in the previous study but maintained them for two weeks following shade removal. In ordinary tea farms, the harvesting of new shoots initiates the emergence of the remaining leaves on the canopy and this begins their exposure to HL illumination (Supplementary Figure S1). Since photosynthetic products generated by the remaining leaves sustain the subsequent growth of tea shoots, the effects of HL stress on the remaining leaves should also be evaluated. Therefore, in this study we designated exposed leaves that remained in the canopy after harvest as “old leaves”, and aimed to elucidate whether or not old leaves experienced photooxidative stress caused by HL exposure in shade-cultivated tea trees.

For this experiment, tea plants (*C. sinensis* (L.) Kunze ‘Yabukita’) grown in an experimental field of Tea Industry Research Division, Agriculture and Forestry Technology Department, Kyoto Prefectural Agriculture, Forestry and Fisheries Technology Center (Uji, Kyoto, Japan; 34°53′N, 135°49′E, Altitude: 84 m) were used in this study. We cultivated tea plants using shade cultivation for eight years (2011 to 2018). During these years, a shading treatment was performed by directly covering the tea canopy for 30 days during the first cropping season and for 20 days during the second cropping season, as described previously (Sano et al. 2020). The current study was then performed in 2017 and 2018, and shading treatments were performed during the periods listed in Supplementary Table S1. In addition, we established non-shaded plots where tea trees were grown under full sunlight to act as experimental controls. Weather conditions (e.g., temperature, sunlight, and precipitation) were monitored throughout the study period using a meteorological observation system (Supplementary Figures S2–S5) as described previously (Morita et al. 2022).

On the day of the completion of the shading treatment (i.e., day 0), Chl fluorescence was measured in shaded leaves just prior to shade removal using a handheld chlorophyll fluorometer (Model OS30p, Opti-Sciences, Inc., NH, USA), using a previously described protocol (Sano et al. 2020). Shade was then removed between 11:00 and 13:15, and new shoots (about 6 cm long) were immediately cropped using a handheld tea harvesting machine (Supplementary Figure S1). New shoots present in control plots were cropped in a similar manner. Old leaves were sampled on the same day for the measurements of Chl and the protein carbonyl content immediately following the harvest of new shoots. The samples for the protein carbonyl assay were stored at −80°C until use. Sampling and Chl fluorescence measurement were also performed between 10:30 and 13:45 at another, later timepoint (1 to 15 days after shade removal).

Next, Chl *a* and Chl *b* content were measured in old leaves using high performance liquid chromatography (HPLC) as per a protocol described by Zapata et al. (2000) with modification. Leaf sampling and Chl extraction in acetone were performed as previously described (Sano et al. 2020). Each extract was then analyzed using an HPLC system (Shimadzu, Kyoto, Japan) that consisted of two chromatographic pumps (LC-10ATvp) to deliver a mobile phase, a system controller (SCL-10ADvp), a mixer (FCV-1AL), a sample injector (SIL-10ADvp), a column oven (CTO-10Avp), a photodiode array detector (SPD-M10Avp), a Symmetry C8 column (3.5 µm, 4.6 mm i.d. ×150 mm; Waters, Milford, MA, USA) and a guard column (SecurityGuard, C8; 3.0 mm i.d. ×4 mm; Phenomenex, Torrance, CA, USA). Chls were detected via monitoring solution absorbance at 400–700 nm. Calibration curves were obtained using standard samples of Chl *a* and Chl *b* purchased from Sigma-Aldrich (St. Louis, MO, USA).

Quantification of protein carbonyls was performed through the reaction of carbonyls with 2,4-dinitrophenylhydrazine, as per a previously described method (Romero-Puertas et al. 2002).

After the second cropping season, yield and yield components were evaluated for new shoots that had emerged in autumn in 2018. To do so, we performed a quadrat survey of six squares (200×200 mm in size) in each plot on 22 October 2018 (i.e., 102 days after the harvest of the second cropping season) as described previously (Morita et al. 2022).

Since the major aim of this study was to investigate the stress response of old leaves to HL in the first and the second cropping seasons, we assessed whether old leaves were stressed by HL illumination after shade removal during 2017 and 2018. We measured Chl fluorescence of photosystem II and monitored changes in Fv/Fm in the old leaves of shade-treated tea trees over a two-week period following the harvest of young shoots (Figure 1). No significant differences in *F*v/*F*m values were observed between control and shaded plots on the day of shade removal. These *F*v/*F*m values decreased significantly in the shaded plot 1–3 days after shade removal but subsequently increased to their initial levels after 7–14 days (except during the second cropping season in 2018) (Figure 1A, B, C). These results indicate that shade-treated old leaves suffered from temporal photoinhibition caused by HL illumination after harvest.

**Figure 1:**
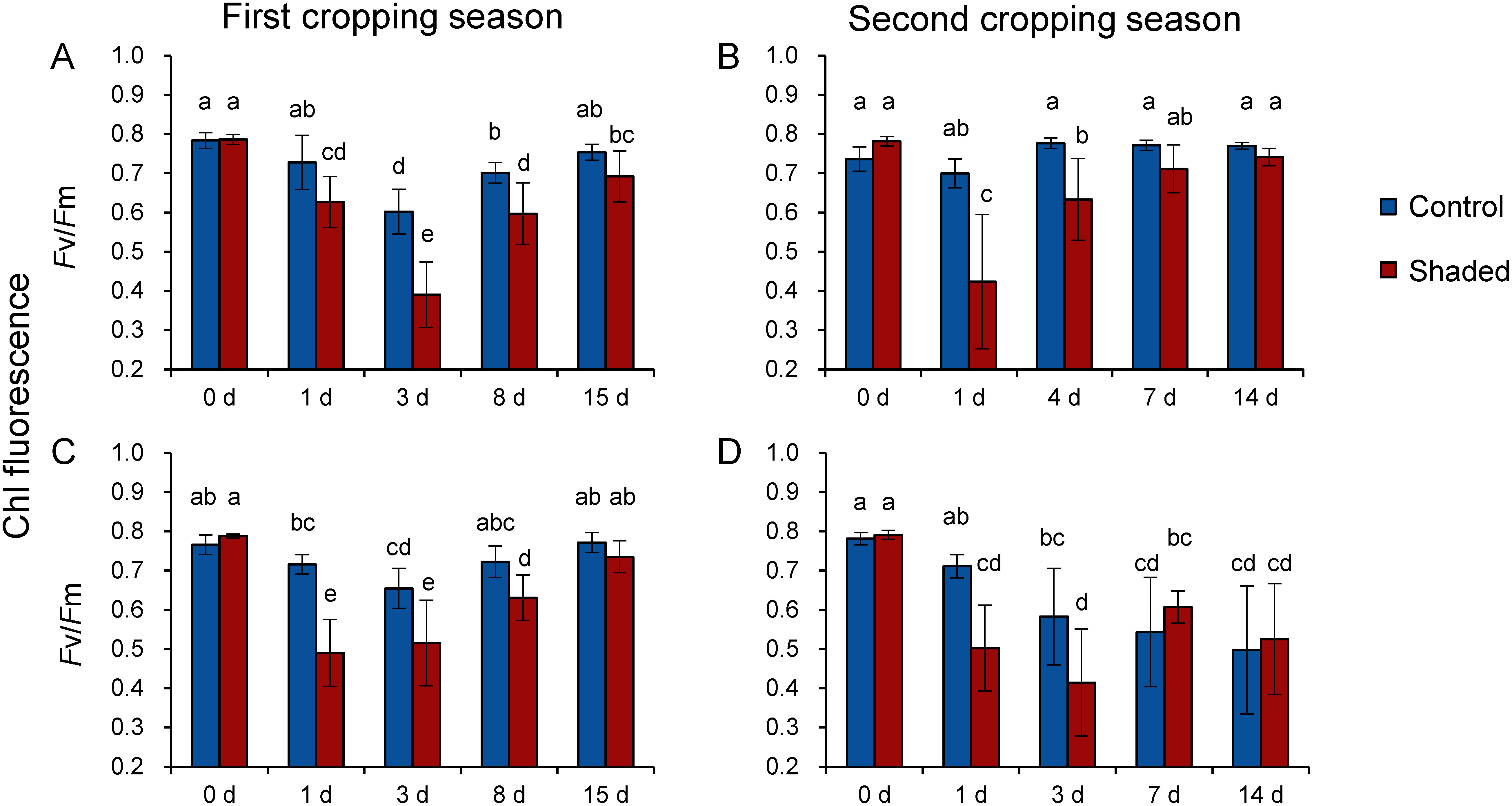
Figure 1. Changes in Chl fluorescence (Fv/Fm) in old leaves following harvest. Data from 2017 (A, B) and 2018 (C, D) are shown as means±standard deviation (*n*=11–16). The x axis indicates days after harvest. Different letters indicate significant differences between samples (*p*<0.05; Tukey–Kramer tests).

Moreover, we also observed a significant decrease in *F*v/*F*m values in old leaves in the control plot on the 3rd day after harvest in the first cropping season, with initial levels being reached after two weeks (Figure 1A, C). These results suggest that old leaves in the control plot were also stressed by HL exposure after harvest. Although tea plants in the control plot were grown without a shading treatment, old leaves were covered with upper leaves before harvest, which might also provide a shade effect. Growing at high planting density can cause the mutual shading of lower leaves and can induce a shading response in field-grown sorghum as evidenced by decreases in leaf thickness and the number of stomata (Li et al. 2014). Therefore, it is likely that old tea leaves grown in the non-shaded plot also acclimated to LL conditions when they were beneath upper leaves. Thus, temporal photoinhibition occurred once they emerged on the canopy and was exposed to HL illumination after harvest.

In the second cropping season of 2018, we found that *F*v/*F*m values decreased 1–3 days after harvest but subsequently increased. However, we determined that they failed to reach their initial levels in both the control and shaded plots (Figure 1D). The recorded maximum daily temperature was around 37°C during the second cropping season of 2018 (Supplementary Figure S5), but it did not exceed 35°C in 2017 (Supplementary Figure S3). Therefore, the fact that the *F*v/*F*m value recorded on the 14th day after harvest was lower than that recorded on day 0 is likely due to a severe heat stress that occurred during the summer season.

Next, we measured Chl content and determined the Chl *a*/*b* ratio of old leaves as an index of the LL/HL response. In general, the Chl *a*/*b* ratio decreases under LL conditions due to increases in the levels of light-harvesting Chl-binding proteins (Leong and Anderson 1984). In this study, we first spectrophotometrically measured the Chl content of old leaves, as done in a previous study (Sano et al. 2020). However, in several samples the Chl *a*/*b* ratio was found to be <2 (data not shown), which was considered to be abnormally low. We speculated that there may be substances contained in old leaves that can affect spectrophotometric measurements, and therefore measured the Chl content of old leaves by HPLC instead. We observed no significant differences in total Chl content between control and shaded plots, except on day 0 of the first cropping season in 2017 (Figure 2B, C, D). In contrast, we also observed that total Chl content decreased significantly in the shaded plot 8–15 days after harvest (Figure 2). Next, we found no significant differences in the Chl *a*/*b* ratios of control and shaded plots on the day 0 and day 7/8 (Figure 3), thereby indicating that Chl *a*/*b* ratio remained unchanged in old leaves in response to LL condition. Taken together, these results suggest that old leaves did not show characteristic LL responses, such as an increase in Chl content and in the Chl *a*/*b* ratio during the shaded treatment. Meanwhile, old leaves decreased total Chl content in response to post-harvest HL exposure.

**Figure 2:**
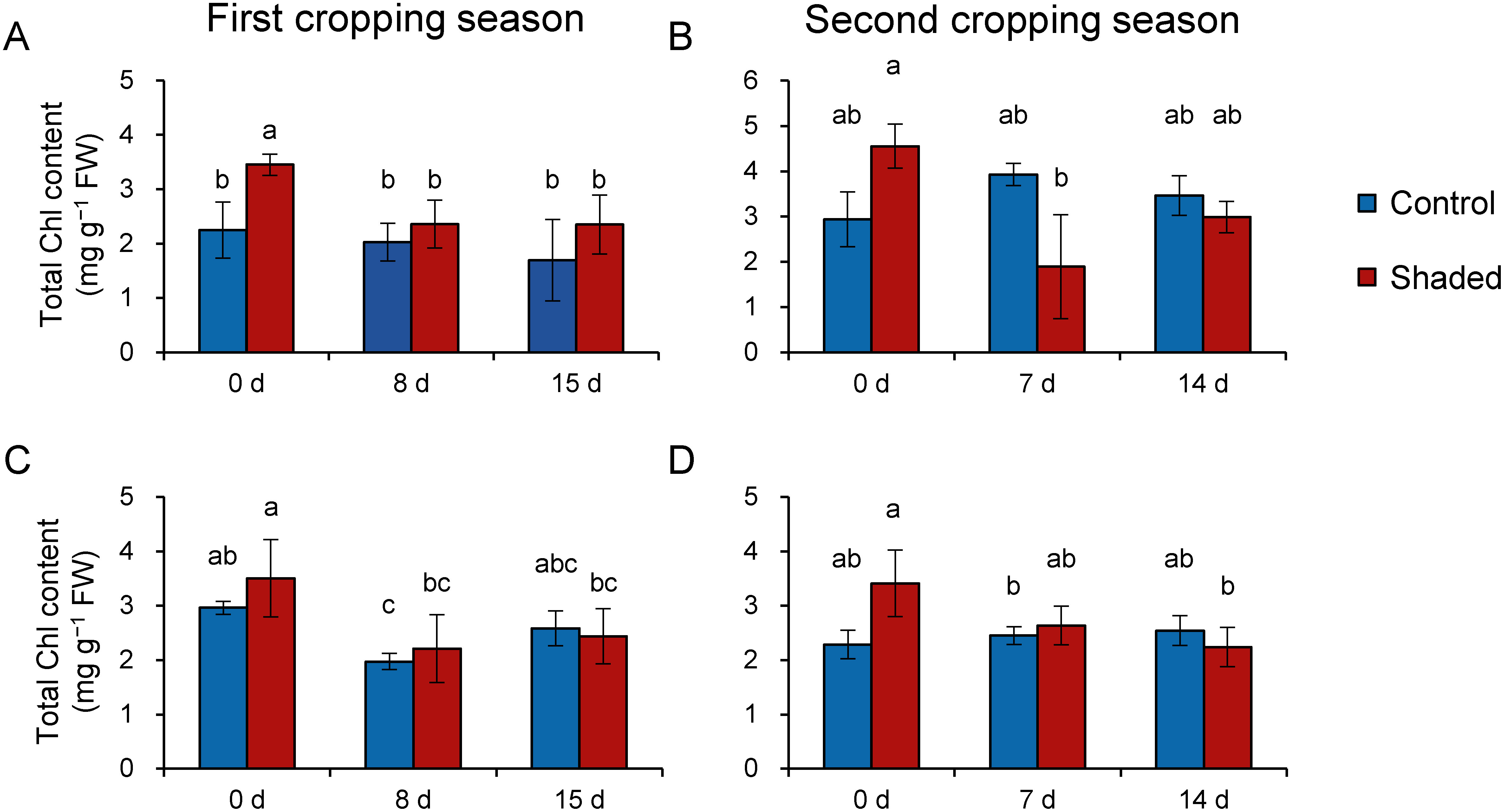
Figure 2. Changes in total Chl content in old leaves following harvest. Data from 2017 (A, B) and 2018 (C, D) are shown as means±standard deviation (*n*=3–5). The x axis indicates days after harvest. Different letters indicate significant differences between samples (*p*<0.05; Tukey–Kramer tests).

**Figure 3:**
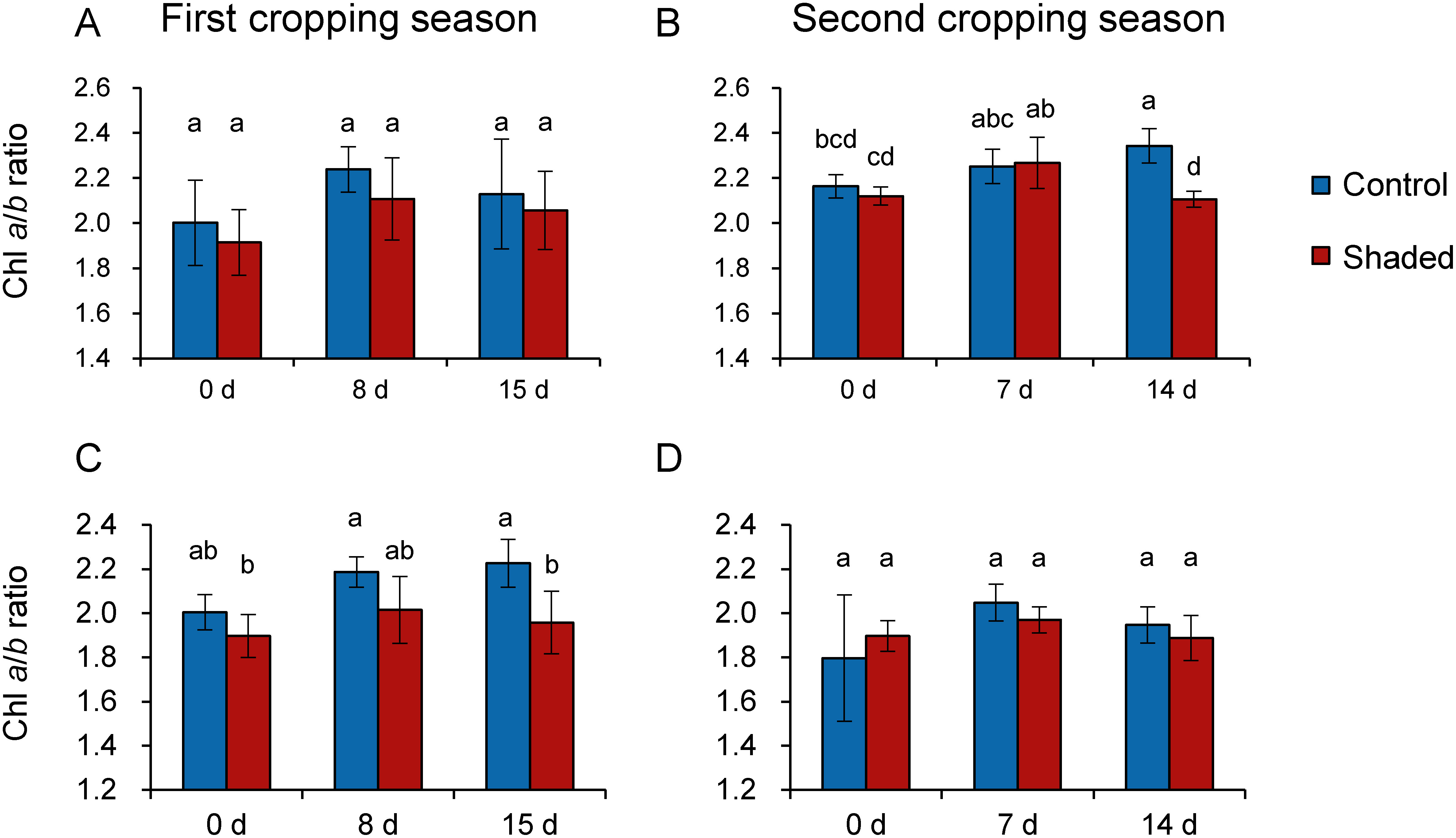
Figure 3. Changes in the Chl *a*/*b* ratio in old leaves following harvest. Data from 2017 (A, B) and 2018 (C, D) are shown as means±standard deviation (*n*=3–5). The x axis indicates days after harvest. Different letters indicate significant differences between samples (*p*<0.05; Tukey–Kramer tests).

To evaluate oxidative injuries provoked by HL illumination, we measured the protein carbonyl content of old leaves (Figure 4). We found no significant changes in the content induced by HL illumination and no differences between shaded and control plots during the first cropping seasons (Figure 4A, C). Moreover, these values remained unchanged after shoot harvesting both in the control and shaded plots as well in the second cropping season of 2017 (Figure 4B). However, we observed different responses during the second cropping season of 2018. We found similar protein carbonyl content values in the shaded and control plots on both day 0 and day 1, but these increased to significantly higher levels in shaded plants than in control plants by days 3–7 (Figure 4D). This result indicated that there was an accumulation of oxidative injuries following HL illumination in shaded old leaves.

**Figure 4:**
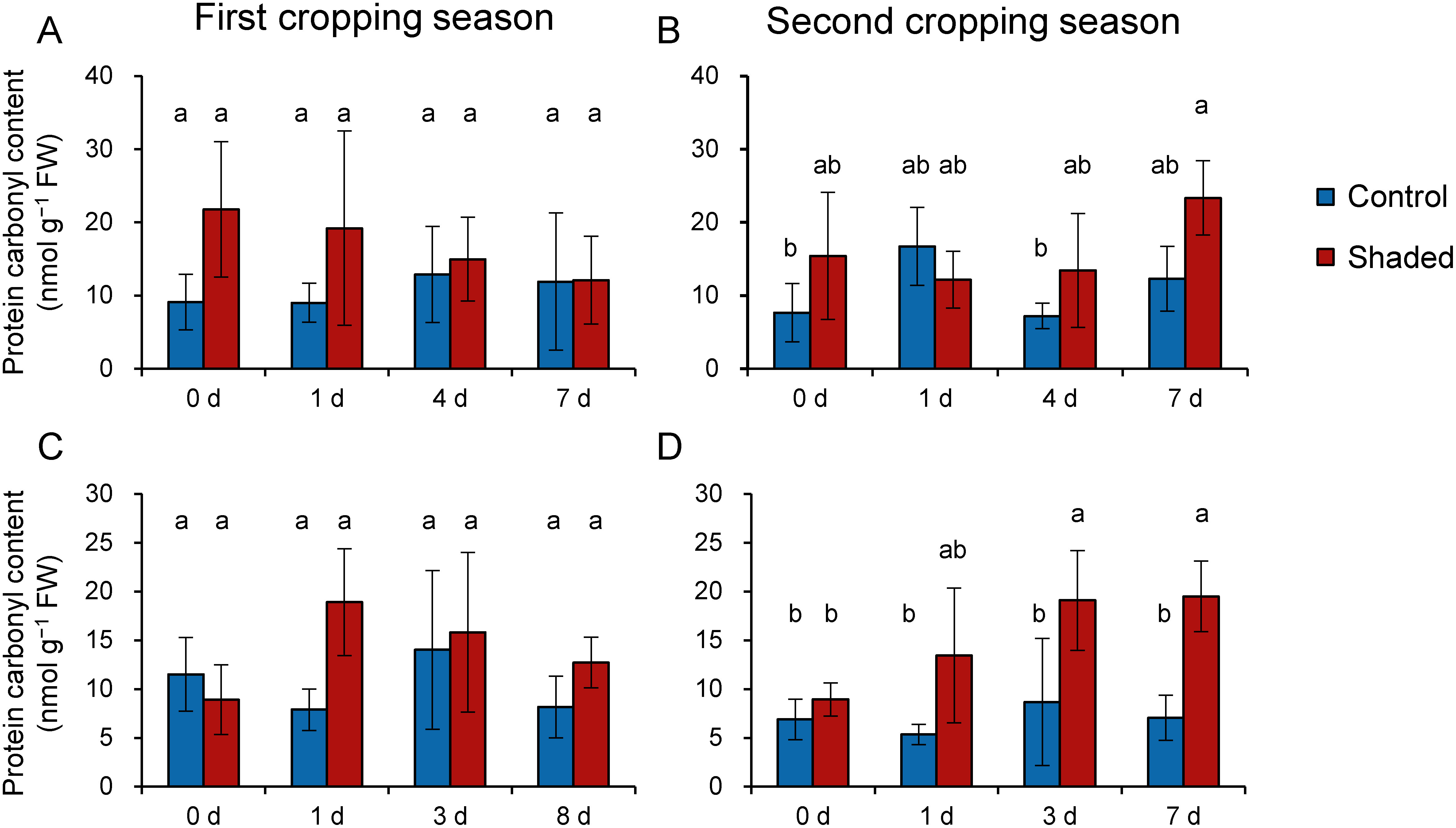
Figure 4. Changes in protein carbonyl content of old leaves following harvest. Data from 2017 (A, B) and 2018 (C, D) are shown as means±standard deviation (*n*=3–5). The x axis indicates days after harvest. Different letters indicate significant differences between samples (*p*<0.05; Tukey–Kramer tests).

We observed different responses in shaded old leaves in the second cropping season between 2017 and 2018, which may be attributed to differences in weather conditions. Cloudy weather predominated during the seven days after shade removal in 2017 (Supplementary Figure S3), whereas the weather was sunny after day 2 in 2018 (Supplementary Figure S5). In addition, the daily maximum temperature was higher during the study period in 2018 than in 2017, as mentioned above. Therefore, it is suggested that a combination of HL illumination and heat stress caused an accumulation of oxidative damage in old leaves.

In a previous study, we observed that the level of protein carbonyls was elevated by HL illumination on the first day in shaded new shoots (Sano et al. 2020). However, no such increse was observed in old leaves in the current study (Figure 4). Furthermore, previous studies have shown that young tea leaves were more sensitive to cold stress and accumulated higher levels of ROS in response to cold stress than mature leaves (Ding et al. 2018; Li et al. 2018). Taken together, these observations and our current results suggest that young leaves are more sensitive to cold and HL stresses than mature leaves. Moreover, the Chl content and Chl *a*/*b* ratio (Figures 2, 3) results indicate that old leaves show no LL response, but no oxidative injuries by HL illumination were observed during the first cropping season in both years and the second cropping season of 2017 (Figure 4A, B, C). Further study is needed to examine how old leaves cope with HL exposure following shoot harvesting.

Our results also revealed the appearance of oxidative injuries in shaded plants in the second cropping season in 2018. We also observed severe leaf damage in old leaves 14 days after harvest in this season (Supplementary Figures S6A, S7A). To quantitatively evaluate leaf damage, we calculated the area of the brown region of the leaf as well as its ratio to total leaf area using image processing of tea canopy photographs (Supplementary Figure S6; Details of the method are described in Supplementary File 1). A comparison of the damaged areas of shaded and control plants revealed significantly more severe injuries in the former (Supplementary Figure S7B).

Next, to investigate whether the foliar damage observed in the second cropping season could affect the growth of tea plants during the subsequent season, we further examined shoot growth during the autumn. We observed that shoot length, the weight of 100 shoots, and the number of new leaves per shoot were lower in the shaded plot than in the control plot, which resulted in a decreased theoretical yield of the shaded plot (Table 1). These results indicate that there was significant suppression in autumn shoot growth in the shaded plot.

**Table 1:** Table 1. Yield components of tea shoots harvested in autumn in 2018.

Experimental plot	Shoot length (cm)	Weight of 100 shoots (g)	Number of new leaves (shoot^−1^)	Number of shoots (400 cm^−2^)	Theoretical yield (kg 10 a^−1^)
Control	2.2±1.3	55.8±6.8	3.2±0.9	67.0±11.7	929±177
Shading	1.4±0.7***	41.3±7.7**	2.8±0.8***	69.3±12.7	702±96*

Data shown are means±standard deviation of six replicates for each plot. Statistical significance was tested by Mann–Whitney *U* test for number of new leaves and *t*-test for rest of the data. Asterisks indicate significant differences between samples (*, *p*<0.05; **, *p*<0.01; ***, *p*<0.001).

Although growth suppression is partially attributable to decreased photosynthetic rates as a direct effect of the shading treatment, this suppression may also be due to damage done to old leaves during the second cropping season. Moreover, it is likely that the suppression of autumn shoot growth can decrease the quantity of photosynthetic products that sustain the growth of new shoots in the next season, which in turn can affect the yield of the first cropping season during the next year. Therefore, alleviation of the damage to old leaves following shade removal is important for avoiding shoot yield loss in the next year. In a recent paper, we found that HL stress in new shoots can be alleviated by acclimating shade-grown tea plants to light prior to shade removal (Morita et al. 2022). Whether the same method can be applicable to ameliorate HL stress in old leaves needs to be addressed by future studies.

In summary, this study revealed that old leaves present on harvested tea plants suffered from temporal photoinhibition after shoot harvesting but were able to recover after two weeks. Furthermore, protein oxidation and severe foliar damage were observed in shade-grown old leaves during the second cropping season, which may result in growth inhibition in autumn shoots. Our results also suggest that the observed damage to shaded old leaves may result in yield reductions in tea shoots that are caused by repeated shade cultivation. Further biochemical and molecular analyses of the stress responses of old leaves and the establishment of cultivation methods capable of alleviating damage to old leaves should be examined further in future studies.

## Abbreviations

Chl: chlorophyll
HL: high light
HPLC: high performance liquid chromatography
LL: low light

## Description of supplementary files

Supplementary File 1. Method for evaluation of leaf damage in tea trees by image processing.

Supplementary Figure S1. Harvest of new shoots and remaining old leaves.

Supplementary Figure S2. Daily temperature, hours of sunlight, and precipitation during the study period in the first cropping season of 2017.

Supplementary Figure S3. Daily temperature, hours of sunlight, and precipitation during the study period in the second cropping season of 2017.

Supplementary Figure S4. Daily temperature, hours of sunlight, and precipitation during the study period in the first cropping season of 2018.

Supplementary Figure S5. Daily temperature, hours of sunlight, and precipitation during the study period in the second cropping season of 2018.

Supplementary Figure S6. Image processing for the quantification of the brown areas of old tea leaves.

Supplementary Figure S7. Damage to old tea leaves after harvest during the second cropping season.

Supplementary Table S1. Shade treatment periods of tea plants in 2017 and 2018.
