# Supplementary material for: Damage in old leaves of shade-treated tea trees induced by high light after shade removal and shoot harvest
Source: Plant Biotechnol (Tokyo). 2025 Mar 25;42(1):105–10. doi: 10.5511/plantbiotechnology.25.0109a (PMC12622896; doi:10.5511/plantbiotechnology.25.0109a)
Supplement: Supplementary Data [file plantbiotechnology-42-1-25.0109a-s001.pdf]

Supplementary File 1

Method for evaluation of leaf damage in tea trees by image processing

Damage to the old leaves present on the canopy of tea trees was quantified by image processing of leaf images taken on the 14th day after harvest in the second cropping season in 2018 (Supplementary Figure S6A). To obtain these images, we set 10 squares (200 × 200 mm in size) on the canopy and took photographs. The resulting RGB images were converted to HSB (hue, saturation, and brightness) stacks using NIH ImageJ (Schneider et al. 2012). Hue and brightness images were then exported separately as text images. Image processing was performed using R (https://www.r-project.org/). To extract the leaf area present within a square, all pixels with a brightness value <50 were deleted, and remaining pixels were regarded as the total leaf area (Supplementary Figure S6B). Brown leaf regions with hue values between 5 to 30 were considered to be damaged leaf area (Supplementary Figure S6C). Next, we counted the number of these pixels and designated it as a damaged area (Supplementary Figure S6D). Finally, the ratio of the damaged area to the total leaf area was calculated for normalization.

References

Schneider CA, Rasband WS, Eliceiri KW (2012) NIH Image to ImageJ: 25 years of image analysis. *Nature Methods* 9: 671-675

Supplementary Table S1. The shade treatment period of tea plants between 2017 and 2018.

Year	Cropping season	Period	Duration
2017	First	April 24 – May 24	30 days
	Second	June 30 – July 20	20 days
2018	First	April 16 – May 16	30 days
	Second	June 22 – July 12	20 days

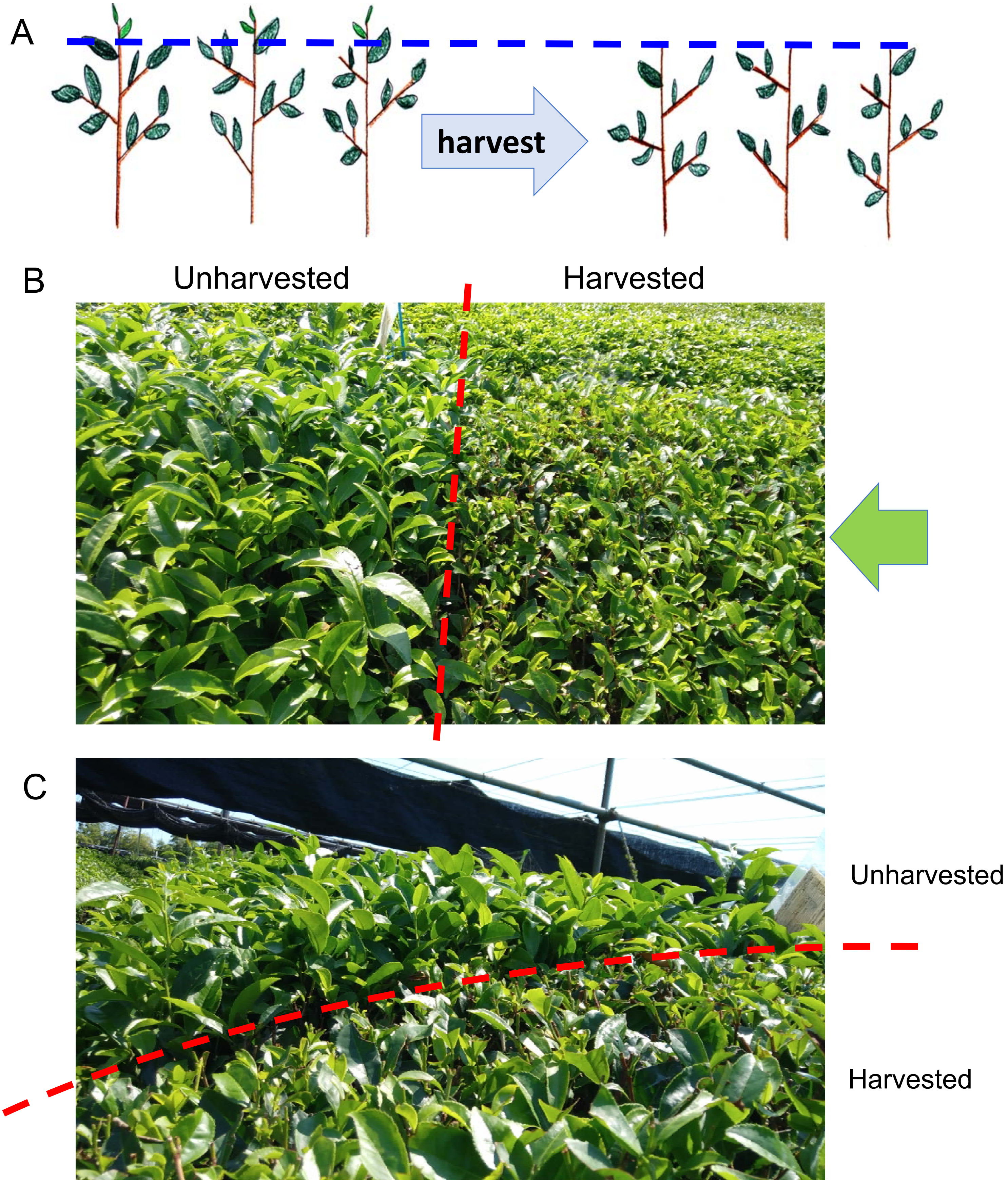

Supplementary Figure S1. Harvest of new shoots and remaining old leaves. (A) Schematic images of the harvest of new shoots. The blue dotted line shows a harvest level and the canopy after harvest. (B) and (C) Photographs of the harvested and unharvested tea trees. Old leaves emerged on the canopy and were exposed to sunlight following harvest. The red dotted line shows a border of the harvested and unharvested area. Panel C shows the same border as shown in B, viewed from the harvested side (depicted in green arrow in panel B). The border line shown in panel C has an arc form as the tea trees used in this study were planted in arc-shaped ridges.

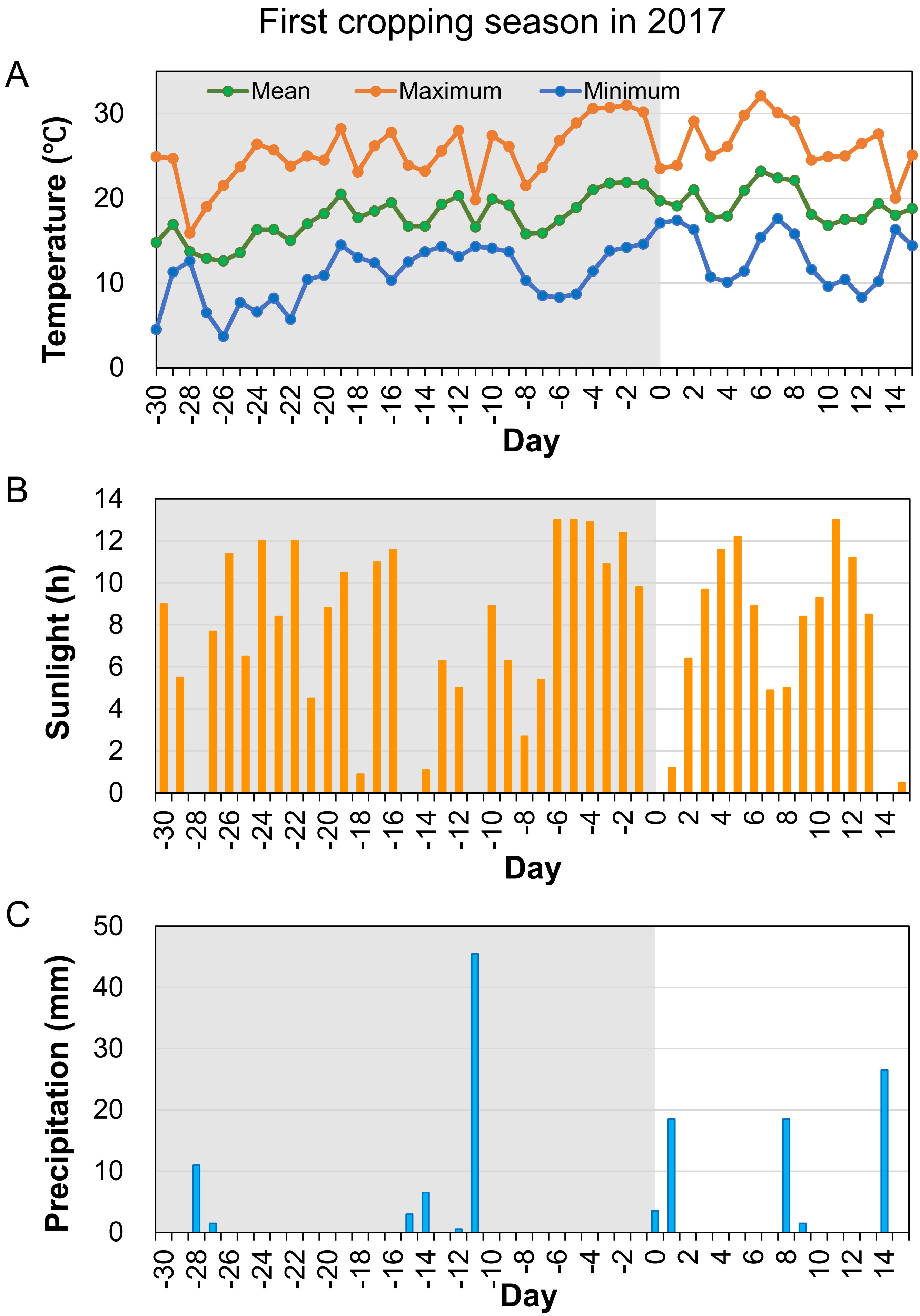

Supplementary Figure S2. Daily temperature (A), hours of sunlight (B), and precipitation (C) during the study period in the first cropping season of 2017. The gray area indicates the period of the shading treatment. The x-axis indicates days after shade removal.

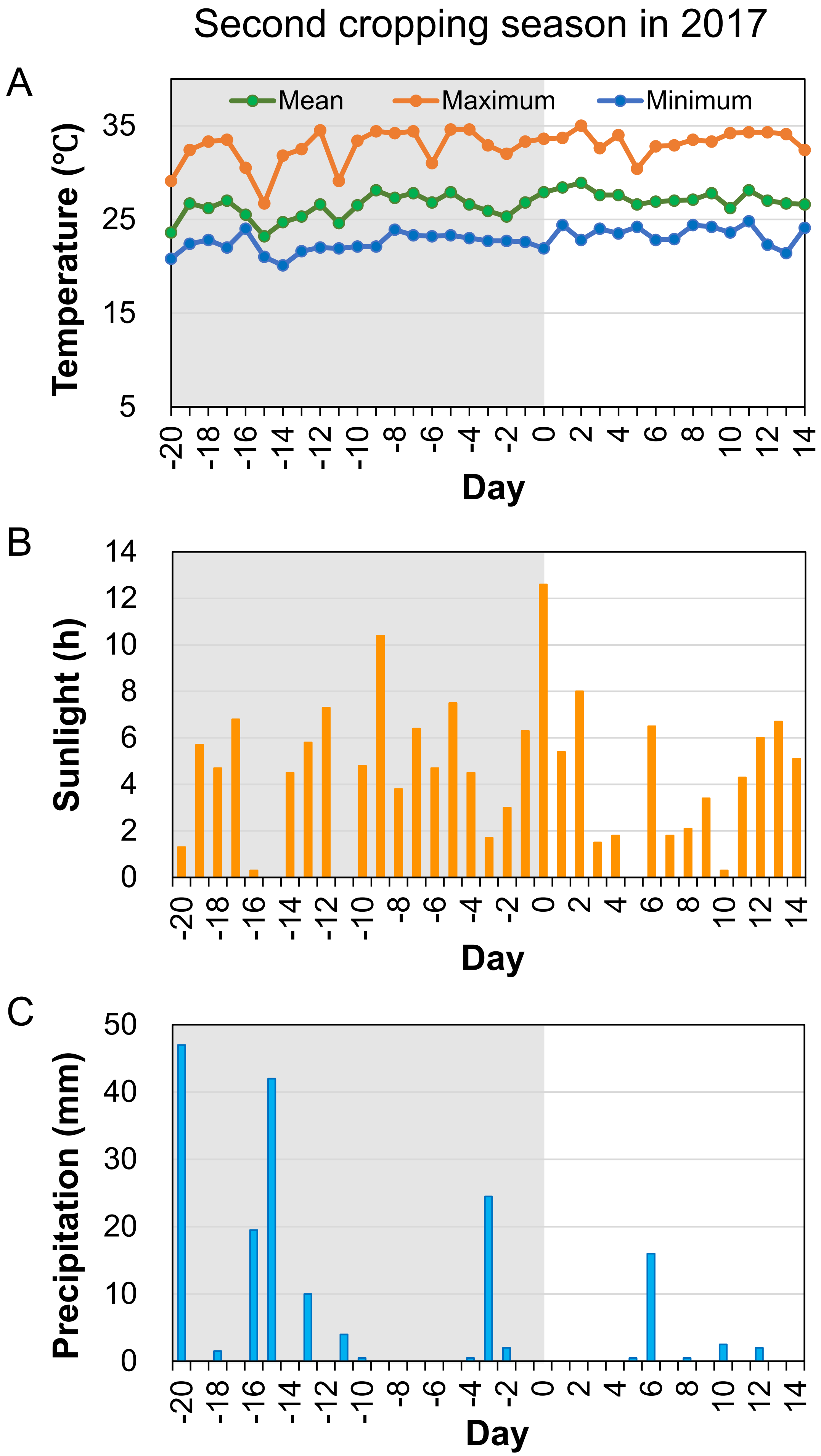

Supplementary Figure S3. Daily temperature (A), hours of sunlight (B), and precipitation (C) during the study period in the second cropping season of 2017.

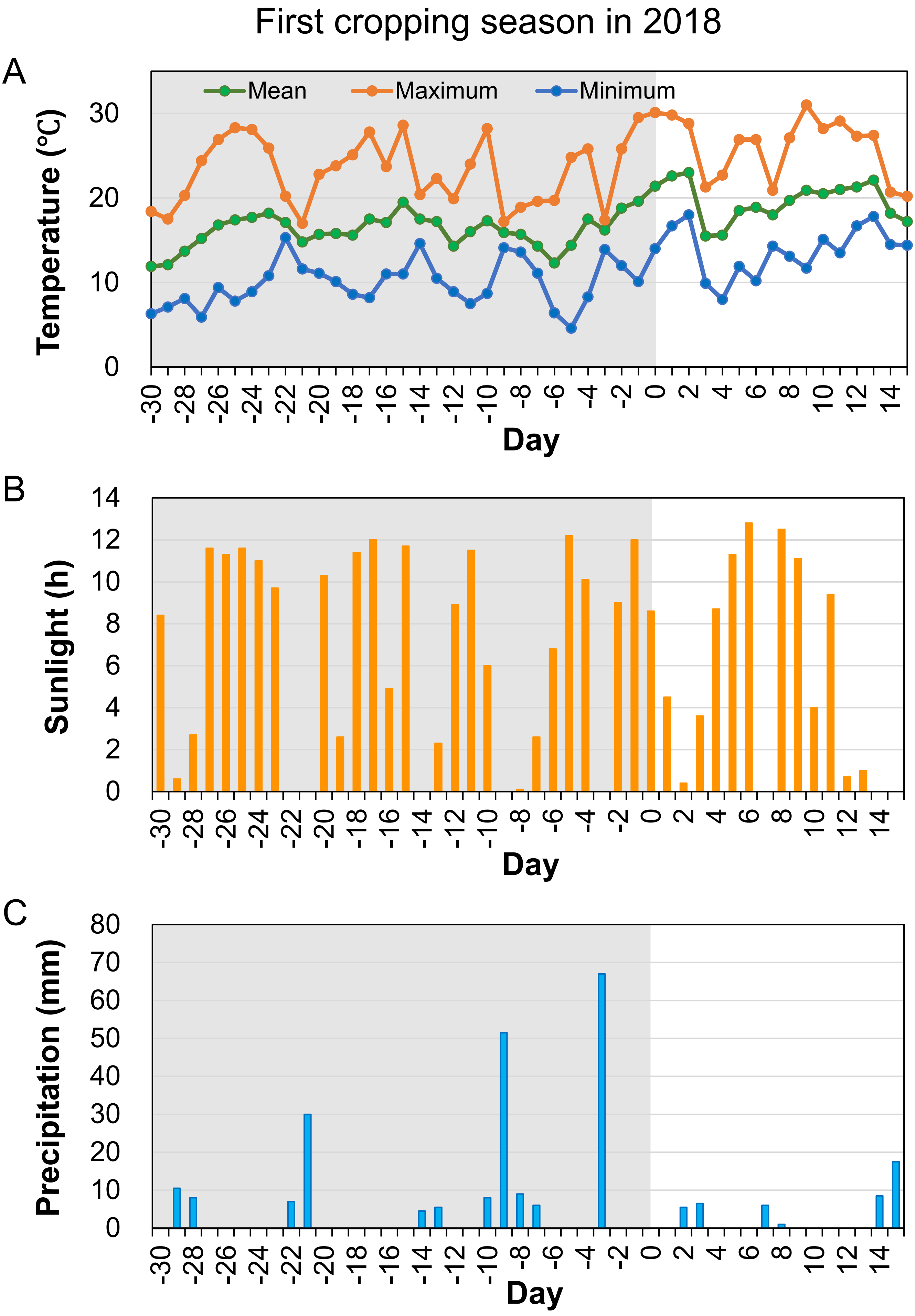

Supplementary Figure S4. Daily temperature (A), hours of sunlight (B), and precipitation (C) during the study period in the first cropping season of 2018.

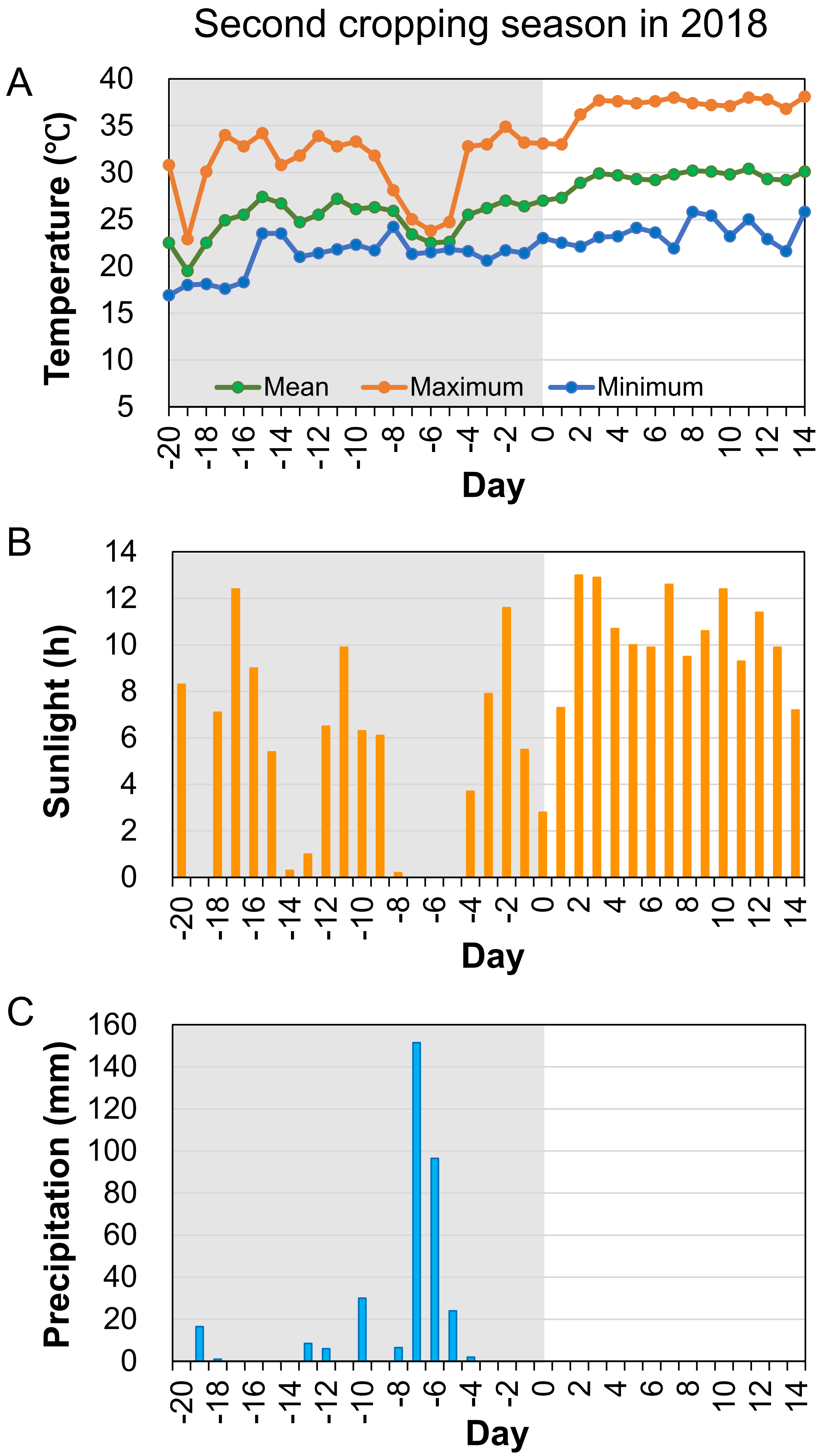

Supplementary Figure S5. Daily temperature (A), hours of sunlight (B), and precipitation (C) during the study period in the second cropping season of 2018.

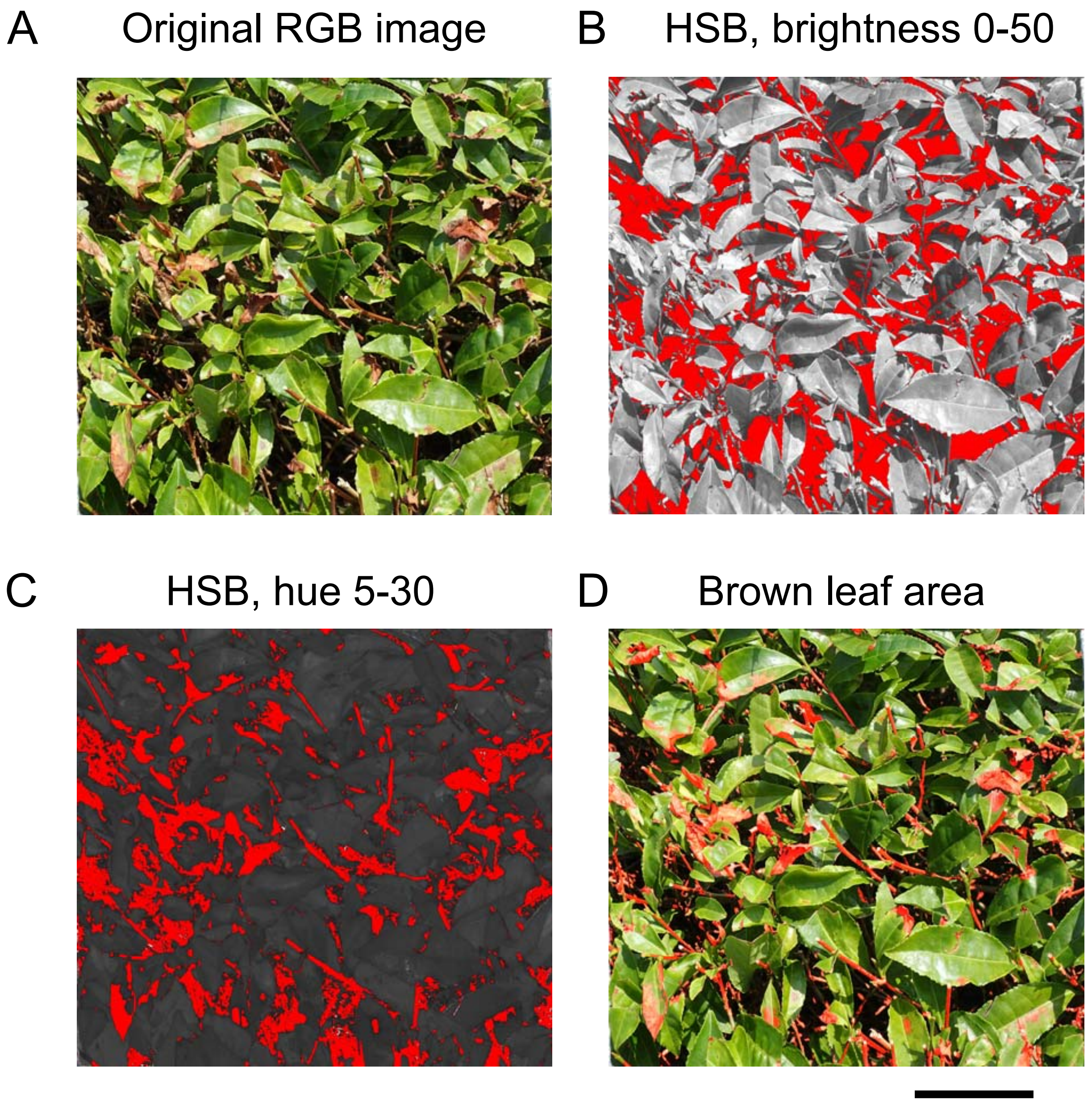

Supplementary Figure S6. Image processing for the quantification of the brown areas of old tea leaves. (A) Original RGB image of a 200 × 200 mm square in size. (B) Leaf area was selected by excluding the pixels with brightness value <50 in the HSB brightness image. The excluded area is shown in red. (C) Brown area was selected by hue level of 5–30, which is shown in red in the HSB hue image. (D) Brown leaf area was defined by selecting the pixels with brightness value ≧50 and hue level of 5–30. This area is shown in red in the RGB image. Scale bar=5 cm.

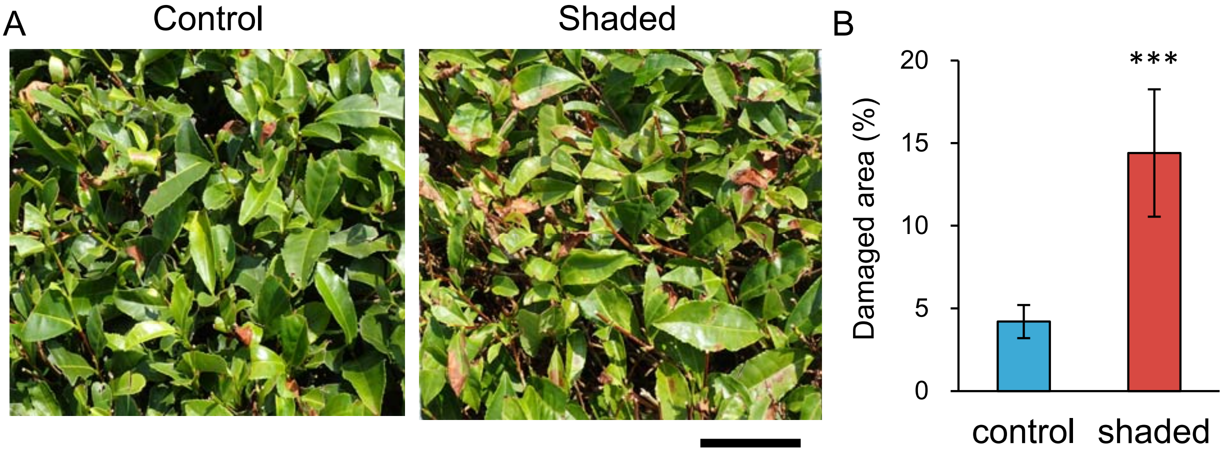

Supplementary Figure S7. Damage to old tea leaves after harvest during the second cropping season. (A) Representative images of the control and shaded plots on the 14th day after harvest in the second cropping season of 2018. Scale bar=5 cm. (B) Comparison of the damaged area on old tea leaves. The ratio of the damaged area to the total leaf area was calculated from the images of the tea canopy. Data are shown as means ± standard deviation ($n = 10$). Asterisks indicate significant differences between the control and shaded plots (***, $p < 0.001$; *t*-test).
